# First Look at the Venoms of Two *Sinomicrurus* Snakes: Differences in Yield, Proteomic Profiles, and Immunorecognition by Commercial Antivenoms

**DOI:** 10.3390/toxins17010019

**Published:** 2025-01-02

**Authors:** Xiang-Yu Li, Ya-Qi Zhang, Xin-Ru Qian, Hong-Yan Zhao, Hong-Liang Lu, Jian-Fang Gao

**Affiliations:** Herpetological Research Center, College of Life and Environmental Sciences, Hangzhou Normal University, Hangzhou 311121, China

**Keywords:** *Sinomicrurus kelloggi*, *Sinomicrurus maccelellandi*, diversity, venom yield, proteome, cross-reaction

## Abstract

Chinese coral snakes (*Sinomicrurus*) are highly neglected regarding their venom profiles and harm to humans, which impedes our ability to deeply understand their biological properties and explore their medicinal potential. In this study, we performed a comparative analysis to reveal the venom profiles of two Chinese coral snakes in terms of their venom yields, proteomic profiles, and immunorecognition by commercial antivenoms. The results showed that *Sinomicrurus kelloggi* expels more venom (lyophilized venom mass) than *Sinomicrurus maccelellandi* but possesses a similar solid venom content. These interspecific differences in venom yield were influenced by the snout–vent length. The venoms of these two species varied in their electrophoretic profiles, as well as in the presence or absence and relative abundance of protein families. They exhibited a 3-FTx-predominant phenotype, where the *S. maccelellandi* venom was dominated by 3-FTx (32.43%), SVMP (23.63%), PLA_2_ (19.88%), and SVSP (12.61%), while the *S. kelloggi* venom was dominated by 3-FTx (65.81%), LAAO (11.35%), and AMP (10.09%). While both the commercial *Naja atra* and *Bungarus multicinctus* antivenoms could immunorecognize these two Chinese coral snake venoms, the *N. atra* antivenom possessed a higher neutralization capability than the *B. multicinctus* antivenom for both species of coral snakes. Our findings show significant interspecific variations in the venom profiles of these *Sinomicrurus* snakes for the first time. We suggest screening or preparing specific antivenoms with high efficiency for the clinical treatment of envenomation caused by these snakes.

## 1. Introduction

Asian coral snakes, also known as Old World coral snakes, are members of elapids with a small–medium size, striking body color, ranging from the tropical to subtropical areas of eastern Asia [1,2,3]. These snakes mainly inhabit forests and roam at night, and they are not frequently encountered by humans, making their mysterious nature difficult to capture. In the past two to three decades, scientists have mainly focused on new records and taxonomies of Asian coral snakes, with many more species being designated or revised, and their suitable habitats have been confirmed to be more extensive [4,5,6,7,8]. Moreover, based on morphological and molecular evidence, Asian coral snakes are further divided into three paraphyletic clades: the tropical mainland clade (current genus *Calliophis*), the northern tropical/subtropical mainland clade (*Sinomicrurus*), and the Philippine clade (*Hemibungarus*) [9]. Currently, Asian coral snakes comprise 28 species (The Reptile Database, accessed on 16 August 2024) [10].

Several investigations have indicated that Asian coral snakes express significant interspecific variations in their venom yields and toxicities. For example, 3-8 mg of dry venom powder per milking can always be collected from the striped coral snake, *C. intestinalis*, but it is non-lethal to mice, frogs, and geckos in vivo, even at high doses [11]. Meanwhile, the blue long-glanded coral snake, *C. bivirgata flaviceps*, can yield 56.63-71.40 mg of dry venom per adult specimen with an LD_50_ of 0.81 µg/g in mice [12]. Nevertheless, Asian coral snakes induce very few envenomation cases and relatively mild symptoms in victims [13,14,15]. So far, only two coral snakes (*C. bivirgata* and *S. macclellandi*) have caused five fatalities in Malaysia, Thailand, Nepal, and Indonesia [16,17]. Asian coral snakes have not yet caused a heavy snakebite burden for human beings. In some Southeast Asian countries, only two Asian coral snakes (*C. bivirgatus* and *C. intestinalis*) have been listed by the WHO in category 2 of medically important venomous snakes for their potential toxicity [18]. Both *C. bivirgatus* and *C. intestinalis* possess 11-12 protein families in their snake venoms, and *C. bivirgatus* contains 14-16 protein families in its venom gland transcripts [11,17,19,20]. The diversity levels of the venom components in these two Asian coral snakes are no less than those in other snakes [21,22,23]. However, the diversity levels of venom components in two other clades of Asian coral snakes, namely, *Sinomicrurus* and *Hemibungarus*, have not been elucidated.

As an important clade of Asian coral snakes, the genus *Sinomicrurus* (also called Chinese coral snake) comprises a total of 10 species, including *S. annularis*, *S. kelloggi*, *S. macclellandi*, *S. peinani*, *S. sauteri*, *S. swinhoei*, *S. boettgeri*, *S. gorei*, *S. iwasakii,* and *S. japonicus*. The first six species are distributed in China [24,25,26]. With the nationwide promotion of biodiversity surveys and monitoring in China, more and more field occurrence records of *Sinomicrurus* snakes have been reported in recent years. In the future, we may have to face the potential increase in snakebite cases caused by *Sinomicrurus* snakes. Moreover, there is currently no commercially available antivenom that has been validated to treat the victims envenomed by these snakes in China. Thus, it is necessary to explore the venom traits of these snakes to facilitate an in-depth understanding of their biological properties and provide a reference for the potential clinical diagnosis and treatment of snakebites caused by them. In this study, we used a small number of adult *S. macclellandi* and *S. kelloggi* specimens for venom collection and employed a combined proteomic strategy of 1D SDS-PAGE and LC-MS/MS to compare the proteomic profiles of these two venoms. Moreover, we evaluated the immunological cross-reactivity between these two Chinese coral snake venoms and heterologous commercial antivenoms in China.

## 2. Results

### 2.1. Morphological Characteristics and Venom Yields

Table 1 shows the morphological characteristics and venom yield profiles of *S. maccelellandi* and *S. kelloggi*. The data on both sexes were combined for the statistical description due to the small sample size (*N* = 4) of each species. One-way ANOVA indicated that all of the morphological characteristics of *S. kelloggi* were larger than those of *S. maccelellandi* (all *p* < 0.01), and *S. kelloggi* (4.9 ± 1.3 mg/per snake) expelled a higher amount of lyophilized venom than *S. maccelellandi* (0.8 ± 0.3 mg/per snake) (*F*_1,6_ = 8.97, *p* = 0.02). Meanwhile, both species showed similar solid venom contents (30.1% for *S. kelloggi* and 31.9% for *S. maccelellandi*; *F*_1,6_ = 0.06, *p* = 0.82). Moreover, one-way ANCOVA revealed no interspecific difference in the amount of lyophilized venom (*F*_1,5_ = 0.003, *p =* 0.96) when using snout–vent length (SVL) as the covariate variable.

### 2.2. Venomic Profiles

**Figure 1:**
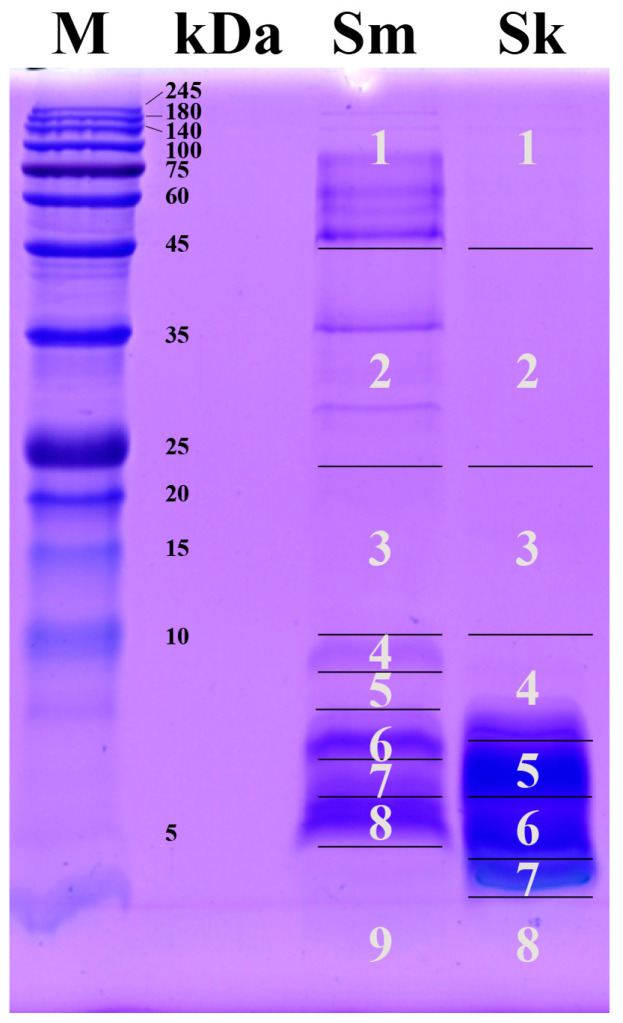
SDS-PAGE profiles of venom proteins from two Chinese coral snakes. Sm, *Sinomicrurus macclellandi*; Sk, *Sinomicrurus kelloggi*. The numerically labeled gel regions were excised, tryptic-digested, and analyzed using nESI-MS/MS. The details of the peptides/proteins are listed in Supplementary Tables S1 and S2.

According to the one-dimensional vertical electrophoretic profiles, twelve and six protein bands were revealed in the *S. maccelellandi* and *S. kelloggi* venoms, respectively. The protein bands of the *S. maccelellandi* venom were mainly located in the molecular weight range of larger than 45 kDa and less than 10 kDa, while those of the *S. kelloggi* venom were mainly located in that of less than 10 kDa (Figure 1). Compared with *S. maccelellandi*, no apparent protein bands were detected in the molecular weight region of larger than 10 kDa in the *S. kelloggi* venom, except for two trace protein bands with molecular weights above 140 kDa. In addition, the relative amount of components with molecular weights of less than 10 kDa in *S. maccelellandi* and *S. kelloggi* accounted for 66.6% and 95.4% of the total venom based on the optical density of the component region in the lanes, respectively (Table 2).

**Figure 2:**
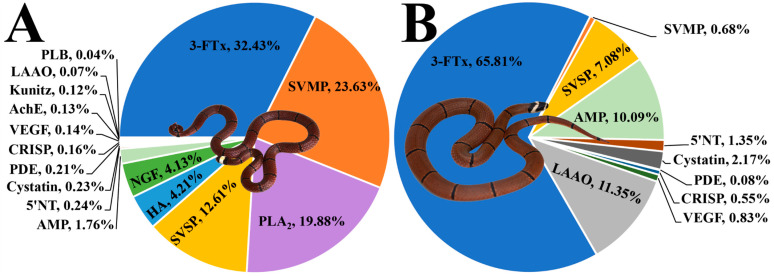
The relative abundances of toxin families in *S. macclellandi* (**A**) and *S. kelloggi* (**B**) venoms. 3-FTx, three-finger toxin; SVMP, snake venom metalloproteinase; PLA_2_, phospholipase A_2_; SVSP, snake venom serine proteinase; HA, hyaluronidase; NGF, nerve growth factor; AMP, aminopeptidase; 5′NT, 5′ nucleotidase; PDE, phosphodiesterase; CRISP, cysteine-rich secretory protein; VEGF, vascular endothelial growth factor; Kunitz, Kunitz-type serine protease inhibitor; AchE, acetylcholinesterase; LAAO, l-amino acid oxidase; PLB, phospholipase B. The details are listed in Supplementary Tables S1 and S2.

The venom proteins in the electrophoretic lanes of these two snakes were sliced into nine and eight gel regions for mass spectrometry identification (Figure 1). The results indicate that the *S. maccelellandi* and *S. kelloggi* venoms comprised 16 and 10 protein families, respectively. Ten of these were shared by both species, including three-finger toxin (3-FTx), snake venom metalloproteinase (SVMP), snake venom serine proteinase (SVSP), aminopeptidase (AMP), 5′ nucleotidase (5′NT), cysteine-type inhibitor (Cystatin), phosphodiesterase (PDE), cysteine-rich secretory protein (CRISP), vascular endothelial growth factor (VEGF), and l-amino acid oxidase (LAAO). Meanwhile, six families were only found in the *S. maccelellandi* venom, including phospholipase A_2_ (PLA_2_), hyaluronidase (HA), nerve growth factor (NGF), acetylcholinesterase (AchE), Kunitz-type serine protease inhibitor (Kunitz), and phospholipase B (PLB) (Figure 2 and Supplementary Tables S1 and S2). Moreover, these two venoms contained six and four protein families with a relative abundance of over 4%, respectively. These relevant families included 3-FTx (32.43%), SVMP (23.63%), PLA_2_ (19.88%), SVSP (12.61%), HA (4.21%), and NGF (4.13%) in the *S. maccelellandi* venom, and 3-FTx (65.81%), LAAO (11.35%), AMP (10.09%), and SVSP (7.08%) in the *S. kelloggi* venom. Significant interspecific variations were found in the relative abundances of several protein families. The relative abundances of 3-FTx, LAAO, and AMP in the *S. kelloggi* venom were 2.0-, 162.1- and 5.7-fold higher than those in the *S. maccelellandi* venom, respectively; meanwhile, the relative abundances of SVMP and SVSP in the *S. maccelellandi* venom were 34.8- and 1.8-fold higher than those in the *S. kelloggi* venom, respectively.

### 2.3. Immunorecognition by Commercial Antivenoms

**Figure 3:**
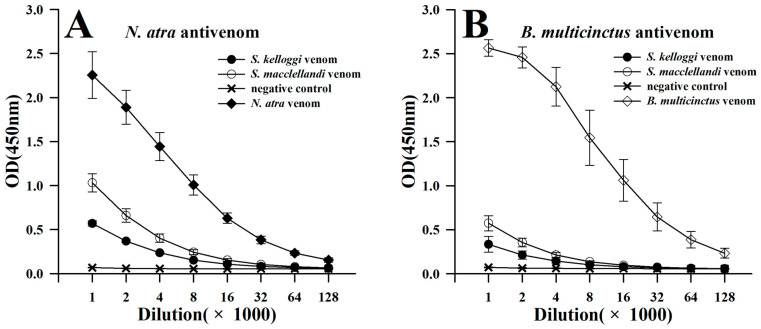
Cross-reaction between Chinese coral snake venoms and two commercial monovalent antivenoms assessed with ELISA. (**A**) *N. atra* antivenom; (**B**) *B. multicinctus* antivenom.

The ELISA test indicated that both commercial monovalent *Naja atra* antivenom and *Bungarus multicinctus* antivenom can immunorecognize *S. maccelellandi* and *S. kelloggi* venoms. The strength of the cross-reaction between venom and antivenom gradually increased with the increase in the concentration of antivenom (Figure 3). These two antivenoms demonstrated a stronger capability to immunorecognize the *S. maccelellandi* venom than the *S. kelloggi* venom. The immunorecognition efficiency for the *S. maccelellandi* venom was nearly 1.8-fold (*N. atra* antivenom) and 1.7-fold (*B. multicinctus* antivenom) higher than that for the *S. kelloggi* venom at a 1:1000 dilution of the antivenoms. Furthermore, the *N. atra* antivenom had a stronger capability to immunorecognize these two Chinese coral snake venoms than the *B. multicinctus* antivenom. The efficiency of the *N. atra* antivenom (OD of *S. maccelellandi*/*S. kelloggi* venom: 1.03/0.57) at a 1:1000 dilution in immunorecognizing both venoms was nearly 1.8- and 1.7-fold higher than that of the *B. multicinctus* antivenom (OD of *S. maccelellandi*/*S. kelloggi* venom: 0.57/0.33). However, these two antivenoms’ heterologous immunorecognition efficiency was much weaker than their homologous immunorecognition efficiency. Specifically, the strength of the cross-reaction between the *N. atra* antivenom and these two Chinese coral snake venoms accounted for 45.7% (*S. maccelellandi*) and 25.2% (*S. kelloggi*) of that with *N. atra* venom (Figure 3A), and only 22.3% (*S. maccelellandi*) and 13.0% (*S. kelloggi*) of that with the *B. multicinctus* venom, respectively (Figure 3B).

**Figure 4:**
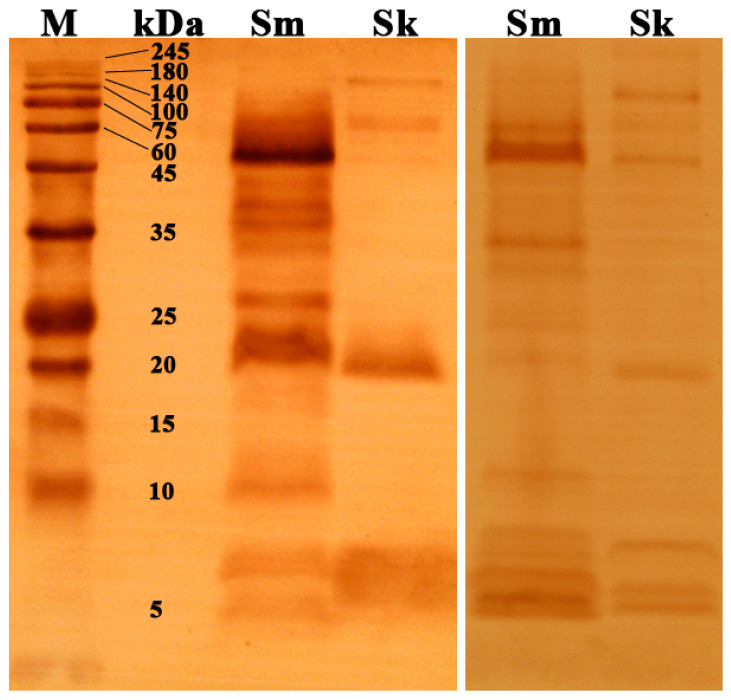
Cross-reaction between Chinese coral snake venoms and two commercial monovalent antivenoms assessed using Western blotting. Left PVDF membrane: venoms incubated with *N. atra* antivenom; right PVDF membrane: venoms incubated with *B. multicinctus* antivenom.

Similarly, Western blotting revealed the difference in the cross-reaction between these two antivenoms and Chinese coral snake venoms, with the reaction regions highly corresponding to the protein bands in the electrophoretogram (Figure 1 and Figure 4). Nearly all of the protein bands in the *S. maccelellandi* venom were immunorecognized by the *N. atra* antivenom, and the region with molecular weights larger than 20 kDa exhibited strong cross-reactivity. Some protein bands with molecular weights of less than 10 kDa in the *S. kelloggi* venom were not immunorecognized, while four trace protein bands larger than 45 kDa were immunorecognized by the *N. atra* antivenom. Moreover, strong cross-reactivity was detected in the component region with molecular weights of approximately 20–25 kDa in both venoms, which was barely found in the electrophoretogram. The components with molecular weights below 10 kDa in both venoms expressed much weaker cross-reactivity with the *N. atra* antivenom compared with those with high molecular weights. Most protein bands of the *S. maccelellandi* venom were immunorecognized by the *B. multicinctus* antivenom, except a few with molecular weights ranging from 35 to 45 kDa. Meanwhile, the *N. atra* antivenom immunorecognized the same protein bands of these two venoms as the *B. multicinctus* antivenom. Overall, the *N. atra* antivenom demonstrated a significantly stronger capability to immunorecognize both *Sinomicrurus* snake venoms than the *B. multicinctus* antivenom.

## 3. Discussion

The snake venom composition from over 200 species has been well interpreted worldwide at the whole-proteome level using a classical strategy combined with multiple technologies, such as RP-HPLC, SDS-PAGE, MALDI-MS/MS, and LC-MS/MS [27]. Among these species, the American coral snake (genus *Micrurus*) accounts for over 10%. Compared with their American relatives in the genus *Micrurus*, Asian coral snakes are highly neglected regarding their venom profiles and harm to humans due to their relatively low encounter rate with humans. This has greatly impeded our progress in better understanding the biological properties and exploring the medicinal potential of Asian coral snakes.

Based on limited studies, the venom yields of different species in the *Calliophis* clade of Asian coral snakes are relatively high and can vary by an order of magnitude. Specifically, up to 15 mg of venom (dry weight) can be yielded from the elongated venom glands of a dissected *C. intestinalis* specimen, and up to an amazing amount of 150 mg can be yielded in a single milking from an adult *C. bivirgata* specimen [11,12,28]. Compared with *Calliophis* snakes, Chinese coral snakes do not have elongated venom glands and can only yield a relatively low amount of venom (as low as 0.8 mg/per snake in *S. maccelellandi*). Moreover, the interspecific variation in venom yield is much lower between these two Chinese coral snakes, with the average venom yield of *S. kelloggi* being approximately 6.1-fold higher than that of *S. maccelellandi*. This is similar to their American relatives in the genus *Micrurus*, with *M. surinamensis* expelling the highest amount (a maximum mean yield of 40.5 mg per snake) of venom and *M. decorates* expelling the lowest amount (a maximum mean yield of 8.8 mg per snake) among the total 15 taxa (with the venom yield varying by less than an order of magnitude between species) [29].

Generally, morphological characteristics are considered to be one cause inducing interspecific and intraspecific variations in venom yield [30,31]. This was validated in the two Chinese coral snakes examined in this study, where their differences in venom yield disappeared after factoring out the potential effect of the SVL. This study only involved a small sample size, which may influence the accuracy of sizes and averages for variations and the significance in statistical results; thus, more samples of both sexes should be collected to validate this conclusion.

The venom proteins of the two Chinese coral snakes were directly separated by 1D SDS-PAGE and identified by LC-MS/MS to elucidate their venomic profiles, as the extremely low venom yield for *S. maccelellandi* made it difficult to collect enough venom for RP-HPLC fractionation. As no venom protein entry for *Sinomicrurus* snakes was found in the reference sequences of serpentes in the UniProt database, the peptides of these two Chinese coral snakes were assigned to known venom proteins in other species based on the sequence similarities. Moreover, this study did not employ absolute quantitative analysis. The relative abundance of each protein was only estimated based on the optical density of the gel region and the spectral intensity of the unique peptide. Overall, these two species significantly varied in terms of their electrophoretic profiles as well as the presence or absence and relative abundance of protein families in the venoms. These two species possessed relatively high diversity levels (8–14 protein families) of venom components, more abundant than those in nearly half of the coral snakes from genera *Micrurus* (3–11 families) [32,33,34,35,36], *Micruroides* (2 families) [37], and *Calliophis* (11–12 families) at the proteomic level [11,17] (Supplementary Table S3). Among these protein families, 3-FTx and PLA_2_ are two critical components [32,33], with a total abundance of over 75% in the venoms of most American coral snakes [34,37] but less than 66% in both the *Calliophis* [11,17] and *Sinomicrurus* (this study) clades of Asian coral snakes.

Asian coral snakes are basal in the phylogeny of coral snakes [38,39], which might imply an evolutionary trend of the total abundance of 3-FTx and PLA_2_ increasing in coral snakes during the dispersal of their ancestral species from the Old World to the New World. On the other hand, a complicated biogeographic distribution pattern of the 3FTx/PLA_2_ venom dichotomy has even been deduced in *Micrurus* snakes [32,33]. Based on this dichotomic system, the venoms of the Chinese coral snakes in this study exhibit a 3FTx-predominant phenotype, while those of *Calliophis* snakes reported in previous studies represent a PLA_2_-predominant phenotype. Thus, the ancestral species of Asian coral snakes may have undergone an evolutionary trend of 3FTx/PLA_2_ venom dichotomy in different clades during the species differentiation process. Considering the low overlap in the distribution areas of the *Sinomicrurus* and *Calliophis* clades [1,2,3], they may have to confront divergent microhabitats as well as local specialized trophic niches. Thus, this dichotomic venom phenotype might be linked to their adaption to their clade-specialized microhabitats and local trophic niches. Nevertheless, this evolutionary trend in the venom phenotype of coral snakes from the Old World to the New World was only inferred based on the putative differences in their regional venom compositions and has not been systematically confirmed in the current study. Therefore, more venomic data on the snakes from the *Sinomicrurus* and *Calliophis* clades, as well as rich quantifiable data on their microhabitats and feeding habits, should be interpreted to validate this speculation.

Antivenom is widely recognized as the most efficient medicine for the clinical treatment of envenomation caused by venomous snakes. The investigation and application of monovalent and polyvalent species-specific antivenoms against American coral snake venoms (mainly the genus *Micrurus*) have been well implemented compared with Asian coral snakes [40,41,42]. However, the neutralization capacity of these species-specific antivenoms against the heterologous snake venoms in the genus *Micrurus* may sometimes be weaker than the antivenoms prepared with the snake venoms from other genera in the Elapidae family [43]. Moreover, the preparation and assessment of broad-specificity antivenom, scFv products and oligoclonal nanobody mixture against American coral snake venoms have also attracted attention [44,45,46].

There is currently no commercially available species-specific antivenom for treating victims envenomed by Asian coral snakes due to the extremely low incidence rate of snakebites caused by Asian coral snakes and the relatively high difficulty of collecting adequate venoms from them to prepare antivenoms. Thus, seeking an alternative, such as commercially heterologous antivenoms against other Elapidae snakes, might be an ideal option. Unfortunately, no suitable heterologous antivenoms have been screened to efficiently neutralize the venoms from the *Calliophis* clade of Asian coral snakes, which might be related to the significant structural differences in key components between these venoms and those used for preparing heterologous antivenoms [11,47].

In mainland China, two monovalent antivenoms against *N. atra* or *B. multicinctus* venom are widely used to treat envenomation caused by common venomous snakes in the Elapidae family [48]. According to the ELISA and Western blotting analysis, these two antivenoms presented a significantly stronger ability to immunorecognize the *S. maccelellandi* venom than the *S. kelloggi* venom. The *N. atra* antivenom also displayed a stronger ability to immunorecognize these two snake venoms than the *B. multicinctus* antivenom. Hence, the *S. maccelellandi* venom probably shares many more common antigens or higher epitope similarity with the *N. atra* and *B. multicinctus* venoms than the *S. kelloggi* venom, and both Chinese coral snake venoms share many more common antigens or higher epitope similarity with the *N. atra* venom than with the *B. multicinctus* venom. Thus, applying commercial *N. atra* antivenom could be a provisional strategy for the clinical treatment of snakebites caused by these two Chinese coral snakes.

However, the intensity of the component regions with an MW of <10 kDa in these two venoms detected using Western blotting was opposite to that using SDS-PAGE. Thus, the structure of the components (MW < 10 kDa) from the same toxin family may differ between these coral snake venoms and the *N. atra* and *B. multicinctus* venoms. As such, these component regions of the Chinese coral snake venoms cannot be efficiently immunorecognized by commercial *N. atra* and *B. multicinctus* antivenoms. Meanwhile, both the *N. atra* and *B. multicinctus* venoms expressed relatively high abundances of the components with low molecular weights (3-FTx and PLA_2_ > 95%) [49], the immunogenicity of which is weaker than those with high molecular weights, resulting in a low abundance of antibodies that immunorecognized the components with low molecular weights in the commercial antivenoms used. These components with MWs of < 10 kDa contained all 3-FTxs (32.43% and 65.81% for *S. maccelellandi* and *S. kelloggi*, respectively) in both venoms and the most PLA_2_s (18.9%) in the *S. maccelellandi* venom (Table 2), which may be the major contributors to the clinical snakebite symptoms caused by these two Chinese coral snakes. However, it has not been evaluated by animal experiments in current study. Accordingly, further evaluations should be conducted (e.g., an evaluation of the protective effects in vivo in a mouse model) before the provisional application of *N. atra* antivenom to treat victims bitten by these two Chinese coral snakes, especially for *S. kelloggi*, which possesses a relatively high venom yield of up to 8.4 mg (lyophilized status) but weak cross-reactivity with commercial antivenoms. Furthermore, attempts should be made to screen candidates with high binding efficiencies to these two Chinese coral snake venoms from other Elapidae commercial antivenoms (e.g., those against *Micrurus* snake venoms) or to prepare scFv products and oligoclonal nanobody mixture with high titers using genetic-engineering techniques.

## 4. Conclusions

We measured the venom yields of *S. macclellandi* and *S. kelloggi* based on a small number of specimens and compared the proteomic profiles of these two venoms with a combined strategy of 1D SDS-PAGE and LC-MS/MS to explore the potential interspecific variation in the venom profiles of Chinese coral snakes. We also evaluated the immunorecognition of these venoms by two heterologous monovalent antivenoms. Specifically, *S. kelloggi* (4.9 ± 1.3 mg/per snake) yielded more lyophilized venom than *S. maccelellandi* (0.8 ± 0.3 mg/per snake), while both species showed similar solid venom contents. Moreover, we validated that the interspecific difference in the venom yield was related to the SVL. These two snakes’ venoms mainly comprised proteins with molecular weights of less than 10 kDa, which accounted for 66.6% and 95.4% of the proteins in *S. maccelellandi* and *S. kelloggi*, respectively. Mass spectrometry analysis revealed that the *S. maccelellandi* and *S. kelloggi* venoms comprised 16 and 10 protein families, respectively, 10 of which were shared by both venoms. These two venoms exhibited a 3FTx-predominant phenotype, where the *S. maccelellandi* venom was mainly dominated by 3-FTx (32.43%), SVMP (23.63%), PLA_2_ (19.88%), and SVSP (12.61%), and the *S. kelloggi* venom by 3-FTx (65.81%), LAAO (11.35%), and AMP (10.09%). Alongside *Calliophis* snakes, based on the PLA_2_-predominant phenotype in the venom, ancestral species of Asian coral snakes were inferred to have undergone an evolutionary trend of 3FTx/PLA_2_ venom dichotomy in different clades during the species differentiation process. Both commercial *N. atra* and *B. multicinctus* antivenoms could immunorecognize these two Chinese coral snake venoms, and the *N. atra* antivenom possessed a higher capability than the *B. multicinctus* antivenom. Nevertheless, the components with low molecular weights in both venoms could not be efficiently immunorecognized by these two antivenoms. Overall, a high level of interspecific variation was identified in the venom profiles of *S. maccelellandi* and *S. kelloggi*, which facilitates an in-depth understanding of their biological properties and provides a reference for the potential clinical diagnosis and treatment of snakebites caused by them.

## 5. Materials and Methods

### 5.1. Animals, Venoms, and Ethics

Four adults of both sexes of each Chinese coral snake (*S. macclellandi* and *S. kelloggi*) with no accurate sampling sites were intermittently collected from four anonymous reptile enthusiasts in mainland China between 2014 and 2022. The snakes were transferred to the Herpetological Research Center at Hangzhou Normal University and maintained individually in 33 cm × 23 cm × 15 cm plastic boxes for venom extraction. Fresh snake venom was extracted and centrifuged according to our previous study [50] and then lyophilized, weighted, pooled, and stored at −80 °C until use. Morphological characteristics, including head length, head width, SVL, tail length, and body mass, were measured as soon as the snakes were transferred to the laboratory. The collection of snakes and the experimental procedures were supervised and approved by the Animal Research Ethics Committee of Hangzhou Normal University (AREC20140311).

### 5.2. Proteomics Analyses

#### 5.2.1. Sodium Dodecyl Sulfate-Polyacrylamide Gel Electrophoresis (SDS-PAGE)

The crude venom powder was re-dissolved in ddH_2_O, and the venom protein content was determined according to Bradford’s method [51]. The venom protein (6 μg) of these two Chinese coral snakes was mixed with a reducing loading buffer, loaded into the wells, and separated using 12% SDS-PAGE. After electrophoresis, the gel was stained with 0.1% Coomassie brilliant blue R-250 (CBB R-250) and imaged using a Umax2100 densitometer gel scanner (Novax Technologies, Taipei, China).

#### 5.2.2. In-Gel Tryptic Digestion

The protein bands in each lane in the gel were excised, washed with ddH_2_O, and then split into small pieces. The gel pieces were transferred to a 1.5 mL tube and destained with 50 mM NH_4_HCO_3_ in 50% acetonitrile (ACN), rinsed with 100% ACN, and dried under vacuum. Subsequently, the gel pieces were swelled and reduced after being incubated with 50 mM dithiothreitol (DTT) in 50 mM NH_4_HCO_3_ at 56 °C for 1 h and alkylated with 0.1 M iodoacetamide (IAA) in 50 mM NH_4_HCO_3_ at room temperature in the dark for 45 min. The pieces were sequentially rinsed with 25 mM NH_4_HCO_3_, 50 mM NH_4_HCO_3_ in 50% ACN, and 100% ACN and dried under vacuum again for 5 min. Then, they were swelled and digested with 25 mM NH_4_HCO_3_ with 0.1 μg/μL of trypsin (MS grade, Promega, Madison, WI, USA) at 37 °C overnight. The reaction system was supplemented with 0.1% trifluoroacetic acid (TFA) in 30% ACN and vibrated in an ultrasonic oscillator for 15 min, and the peptide-containing solution was collected. The process was repeated, and the peptide mixture in the solution was mixed and lyophilized. Before mass spectrometry identification, the peptide mixture was re-dissolved in 0.1% TFA, desalted using a Millipore^®^ C18 ZipTip (Merck, Darmstadt, Germany), and lyophilized. The peptide mixture was re-dissolved and vibrated in 0.1% formic acid (FA) and 5% ACN and then centrifuged at 13,500 rpm at 4 °C for 15 min, and the supernatant was collected.

#### 5.2.3. Identification of Venom Proteins in Peptide Mixture

The supernatant (8 μL) was separated at a flow rate of 400 nL/min with an EASY-nanoLC 1000 system (ThermoFisher, Waltham, MA, USA) using an Acclaim™ PepMap™ RSLC 100 C18 column (NanoViper; 75 μm × 15 cm, 2 μm; ThermoFisher, Waltham, MA, USA) based on the mobile phase A of 0.1% FA in water and B of 80% ACN in 0.1% FA as follows: 3% B for 3 min, 3–8% B for 4 min, 8–32% B for 34 min, 32–44% B for 5 min, 44–99% B for 5 min, and 99% B for 4 min. The eluted peptides were subjected to a Q Exactive Orbitrap system (ThermoFisher, Waltham, MA, USA) with Electrospray Ionization (ESI) source for identification. ESI source was operated in positive ionization modes with spray voltages of 3.5 kV and the capillary temperature of 320 °C. The mass spectrometer analysis was conducted in full scan/dd-MS2 positive ion mode, and scanned from *m*/*z* 300 to 1400 with the resolution in MS1 and MS2 fixed at 70,000 and 17,500, respectively. The 20 most abundant precursor ions were selected for fragmentation by high collision dissociation (HCD) with normalized collision energy of 27 and isolation window 1.6 *m*/*z*. The dynamical exclusion was set as follows: charge exclusion, unassigned 1, 8, and > 8; peptide match, preferred; exclude isotopes, on; dynamic exclusion, 10 s. The raw MS/MS spectra were interpreted using Xcalibur, and the sequence similarity analysis of the amino acids in the MS/MS spectra was performed using PEAKS X (Bioinformatics Solutions Inc., Waterloo, ON, Canada) against the UniProt database (serpentes, 3,361,556 entries, downloaded on 14 July 2023) (Supplementary Figure S1). The false discovery rate (FDR) of all searches was set at 0.1%. The mass tolerance for the precursor and fragment ion was set at 15 ppm and 0.05 Da, respectively. Carbamidomethyl (C) and oxidation (M) were set as fixed and variable modifications, respectively.

#### 5.2.4. Estimation of Relative Abundances of Venom Proteins

The relative abundances of the venom proteins were estimated relying on the optical densities of the proteins in the gel electropherogram and the spectral intensity of the unique peptides in the MS/MS analysis. Specifically, the optical density of each gel region (Figure 1) was measured using the ImageJ software (1.53e) (National Institutes of Health, Bethesda, MD, USA), and the relative abundance of each region was defined as the proportion of its optical density to the accumulated optical density of all regions in the relevant lane. The relative abundance of each protein family identified in the gel region was similarly defined as the proportion of its unique peptide spectral intensity to the accumulated spectral intensity of all unique peptides in this gel region. Briefly, the ratio of the venom protein was calculated with the following formula: relative abundance of each venom protein in the gel region (%) = (optical density of each gel region/accumulated optical density of all protein regions in the lane × 100) × (spectral intensity one protein/accumulated spectral intensity of all proteins in relevant gel region × 100). Finally, the relative abundance of the same protein family was accumulated to describe the venom’s proteomic profile.

### 5.3. Immunorecognition by Commercial Antivenoms

Enzyme-linked immunosorbent assay (ELISA) was used to evaluate the efficiency of two commercial antivenoms in immunorecognizing the venoms from these two Chinese coral snakes. Specifically, each well in a 96-well microplate was coated with 2 μg/mL of the venom proteins in 100 μL of 0.1M Na_2_CO_3_-NaHCO_3_, pH 9.6 at 37 °C for 2 h. The plate was rinsed thoroughly with PBST (0.05% Tween-20 in 10 mM PBS, pH 7.4) three times to remove the unbound venom proteins. Then, each well was incubated with 150 μL of 2% non-fat milk powder in PBST at 37 °C for 1 h. Subsequently, the plate was rinsed three times again, and each well was incubated with 100 μL of serially diluted monovalent *B. multicinctus* antivenom and *N. atra* antivenom (Shanghai Serum Bio-technology Co., LTD., Shanghai, China) with an initial concentration of 4 μg/μL in PBST and 1% BSA at 37 °C for 1 h. Ordinary horse serum was used as the negative control. The unbound antivenom was washed off, and 100 μL of a secondary antibody (HRP-labeled anti-horse IgG, Sigma, Saint Louis, MO, USA) diluted with PBST and 1% BSA at 1:13,500 was added to each well and incubated at 37 °C for 1 h. Afterward, the microplate wells were rinsed thoroughly with PBST, supplemented with 100 μL of a substrate solution (0.5 mg/mL TMB and 0.006% H_2_O_2_ in 0.15 M citrate buffer, pH 5.0) and incubated at room temperature for 20 min. Finally, 50 μL of 2.5 M sulfuric acid was added to stop the reaction, and the absorbance was auto-recorded at 450 nm using a Varioskan Flash reader (ThermoFisher, Waltham, MA, USA).

The immunorecognition of both two venoms by the commercial antivenoms was performed using Western blotting. The venom proteins were separated by 12% SDS-PAGE under reducing conditions, according to Laemmli’s method [52]. After electrophoresis, the proteins on the gels were either stained with CBB R-250 or transferred to a PVDF membrane (0.45 μm, Millipore, Cork, Ireland) on a Trans-Blot SD transfer cell (Bio-Rad, Hercules, CA, USA). The membrane was blocked with 15% non-fat milk powder in 20 mM TBST at 4 °C overnight, washed thoroughly, and incubated with either monovalent *B. multicinctus* antivenom or *N. atra* antivenom (diluted with 20 mM TBST with 2% BSA at 1:1000) at 37 °C for 1 h. After washing, the membrane was immersed in a secondary antibody solution (AP-labeled anti-horse IgG, Sigma, Saint Louis, MO, USA) diluted with 20 mM TBST containing 2% BSA at 1:3000 and incubated at 37 °C for 1 h. Subsequently, the secondary antibody was washed off thoroughly, and the membrane was immersed in a substrate solution (0.3 mg/mL NBT and 0.15 mg/mL BCIP in 0.1 M Tris-HCl, pH 9.5). Finally, the membrane was transferred to ddH_2_O to stop the reaction and imaged using a Umax2100 densitometer gel scanner. Then, the profiles of the chromogenic bands were analyzed with the ImageJ software.

### 5.4. Statistical Analyses

The description of the morphological characteristics and profiles of the venom yields (lyophilized venom and solid venom content [30]) of the two Chinese coral snakes was performed using Statistica 8.0 (StatSoft Inc., Tulsa, OK, USA). We used one-way ANOVA and one-way ANCOVA to examine the interspecific variations in the morphological characteristics and profiles of the venom yield. The significance level was set at *α* = 0.05.

## Figures and Tables

**Table 1:** Morphological characteristics and profiles of venom yields of two Chinese coral snakes.

	*S*. *macclellandi*	*S*. *kelloggi*	Statistical Results
Sample (*N*)	4	4	
Head length (mm) *	12.5 ± 0.8 11.0–14.6	20.0 ± 0.4 19.1–21.1	*F*_1,6_ = 68.38, *p* = 0.0002
Head width (mm) *	6.5 ± 0.6 5.4–7.9	11.1 ± 0.7 9.3–12.4	*F*_1,6_ = 27.33, *p* = 0.002
Snout-vent length (cm) *	43.8 ± 2.3 38.8–49.8	61.8 ± 1.6 56.9–63.6	*F*_1,6_ = 40.33, *p* = 0.0007
Tail length (cm) *	4.9 ± 0.4 4.0–5.9	7.4 ± 0.4 6.7–8.3	*F*_1,6_ = 20.90, *p* = 0.004
Body mass (g) *	14.9 ± 3.3 8.3–22.8	51.7 ± 7.9 39.9–74.6	*F*_1,6_ = 17.96, *p* = 0.005
Venom yield (mg) *	0.8 ± 0.3 0.2–1.5	4.9 ± 1.3 2.5–8.4	*F*_1,6_ = 33.62, *p* = 0.02
Solid content (%) *^,^ **	30.1 ± 3.7 25.0–40.5	31.9 ± 6.3 15.5–43.5	*F*_1,6_ = 0.06, *p* = 0.82

* Data are expressed as the mean value ± SE and ranges. ** Solid content (%) is calculated from the lyophilized venom mass divided by the fresh venom mass × 100.

**Table 2:** Overview of relative abundances and protein families in gel regions in SDS-PAGE of venom proteins from two Chinese coral snakes. Assignment of sequence similarity in protein identification relied on the UniProt database (strict to the taxa Serpentes).

*S. macclellandi*			*S. kelloggi*		
Gel Region	Relative Abundance	Protein Families	Gel Region	Relative Abundance	Protein Families
1	20.74%	AchE, AMP, HA, LAAO, PDE, SVMP, SVSP	1	1.39%	5′NT, AMP, SVMP, and SVSP
2	10.64%	5′NT, NGF, PLB, SVMP, and SVSP	2	1.70%	5′NT, AMP, SVMP, and SVSP
3	2.02%	CRISP, NGF, and SVMP	3	1.51%	CRISP, SVSP, and VEGF
4	4.00%	NGF, PLA_2_, SVMP, and SVSP	4	12.49%	3-FTx, AMP, LAAO, and SVSP
5	0.80%	NGF, PLA_2_, SVMP, and SVSP	5	39.81%	3-FTx and Cystatin
6	21.65%	3-FTx, Cystatin, and PLA_2_	6	23.78%	3-FTx and SVSP
7	11.36%	3-FTx, PLA_2_, SVMP, SVSP, and VEGF	7	19.17%	AMP, LAAO, and SVSP
8	27.85%	3-FTx, CRISP, Kunitz, NGF, PLA_2_, SVMP, and SVSP	8	0.15%	PDE and SVSP
9	0.95%	SVSP	-	-	

## Data Availability

The original contributions presented in this study are included in the article/Supplementary Material. Further inquiries can be directed to the corresponding authors.

## Supplementary Materials

The following supporting information can be downloaded at: https://www.mdpi.com/article/10.3390/toxins17010019/s1. Table S1: Protein identification of venom from *Sinomicrurus macclellandi* by mass spectrometry; Table S2: Protein identification of venom from *Sinomicrurus kelloggi* by mass spectrometry; Table S3: Summary of estimated relative abundances (%) of protein family in venoms from coral snakes; 3-FTx, three finger toxin; SVMP, snake venom metalloproteinase; PLA_2_, phospholipase A_2_; SVSP, snake venom serine proteinase; HA, hyaluronidase; NGF, nerve growth factor; AMP, aminopeptidase; 5′NT, 5′ nucleotidase; PDE, phosphodiesterase; CRISP, cysteine-rich secretory protein; VEGF, vascular endothelial growth factor; AchE, acetylcholinesterase; LAAO, l-amino acid oxidase; PLB, phospholipase B; CTL, C-type lectin-like; VF, venom factor; BIP, bradykinin inhibitory peptide; ChE-like, Cholinesterase-like protein; Figure S1: Interpretation of peptides “TTATDIK” (A; assigned to NGF in region 4 of *S. macclellandi* venom) and “EVTVDVGK” (B; assigned to VEGF in region 3 of *S*. *kelloggi* venom) from MS spectra.
