# Supplementary figures and images for: First Look at the Venoms of Two Sinomicrurus Snakes: Differences in Yield, Proteomic Profiles, and Immunorecognition by Commercial Antivenoms
Source: Toxins (Basel). 2025 Jan 2;17(1):19. doi: 10.3390/toxins17010019 (PMC11769021; doi:10.3390/toxins17010019)

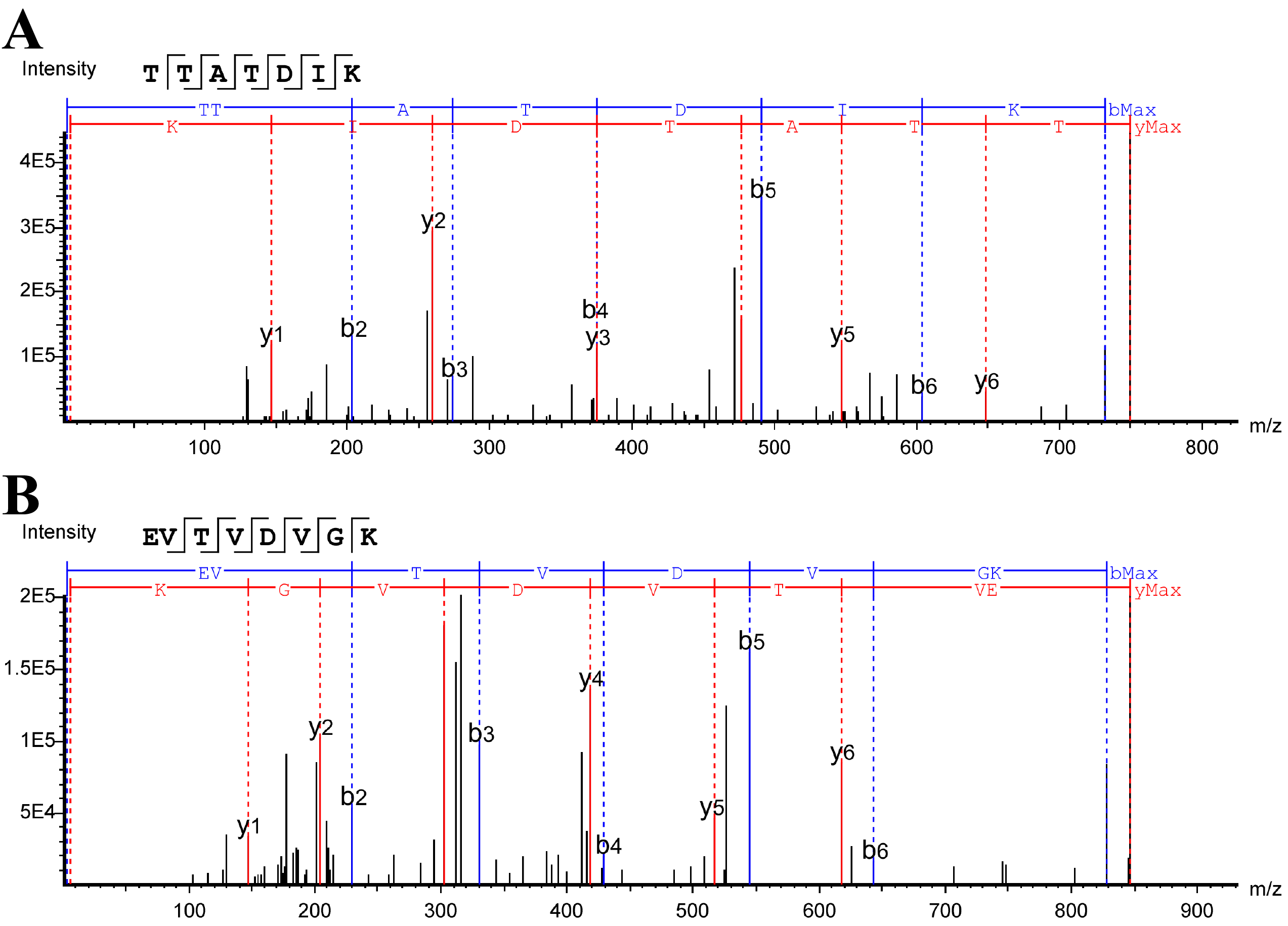

Supplement: Supplementary file 1 [file toxins-17-00019-s001.zip › Supplementary figure S1.png]
